# The Influence of Autoimmune Thyroid Diseases on Viral Pneumonia Development, Including COVID-19: A Two-Sample Mendelian Randomization Study

**DOI:** 10.3390/pathogens13020101

**Published:** 2024-01-24

**Authors:** Kexin Yi, Mingjie Tian, Xue Li

**Affiliations:** 1 School of Public Health, Shanghai Jiao Tong University School of Medicine, Shanghai 200025, China; yikexin1999@sjtu.edu.cn; 2 Shanghai Deji Hospital, Qingdao University, Shanghai 200331, China; tianmj@shneuro.org

**Keywords:** autoimmune thyroid disease, thyroid function, viral pneumonia, COVID-19, Mendelian Randomization

## Abstract

The association between thyroid function and viral pneumonia has undergone extensive examination, yet the presence of a causal link remains uncertain. The objective of this paper was to employ Two-Sample Mendelian Randomization (MR) analysis to investigate the connections between three thyroid diseases and thyroid hormone indicators with viral pneumonia and COVID-19. We obtained summary statistics datasets from seven genome-wide association studies (GWASs). The primary method used for estimating relationships was inverse-variance weighting (IVW). In addition, we employed weighted median, weighted mode, MR-Egger, and MR-PRESSO as supplementary analytical tools. Sensitivity analyses encompassed Cochran’s Q test, MR-Egger intercept test, and MR-PRESSO. Our study revealed significant causal relationships between having a genetic predisposition to autoimmune thyroid disease (AITD) and an increased susceptibility to viral pneumonia (odds ratio [OR]: 1.096; 95% confidence interval [CI]: 1.022–1.176). Moreover, it demonstrated a heightened susceptibility and severity of COVID-19 (OR for COVID-19 susceptibility, COVID-19 hospitalization, and COVID-19 critical illness, with 95% CIs of 1.016, 1.001–1.032; 1.058, 1.003–1.116; 1.045, 1.010–1.081). However, no statistically significant associations were found between TSH, FT4, subclinical hypo- or hyperthyroidism, and the risk of viral pneumonia incidence, or the susceptibility and severity of COVID-19 (all *p* > 0.05). This study establishes a cause-and-effect relationship between AITD and the development of viral pneumonia, as well as the susceptibility and severity of COVID-19.

## 1. Introduction

Viral pneumonia, particularly COVID-19 resulting from SARS-CoV-2 infection, has exerted a profound impact on both human health and socio-economic development [1,2,3,4,5,6], rendering it a paramount global public health concern [7,8]. Identifying susceptible risk factors and establishing their causal relationships is imperative to mitigate the incidence of these diseases.

Current evidence suggests that the thyroid gland is a potential target organ for viral infection [9,10,11]. Thyroid dysfunction has been demonstrated to detrimentally affect lung protective mechanisms [12]. Moreover, a close correlation exists between thyroid dysfunction, metabolic disorders, and intricate interactions with the immune system [13,14,15,16,17]. These immune dysregulations have unequivocally been associated with the onset and progression of viral pneumonia [1,2,18]. It can be inferred that inadequate management of potential thyroid disorders may heighten the risk and severity of viral pneumonia. Nevertheless, epidemiological studies on the causality between thyroid diseases or hormones and viral infection-induced pneumonia remain scarce [19,20].

Among the various types of viral pneumonia, extensive research has focused on comprehending the underlying mechanisms of COVID-19. SARS-CoV-2 targets the thyroid and the hypothalamic–pituitary–thyroid axis (HPT). The expression of SARS-CoV-2 cell receptor and angiotensin-converting enzyme 2 (ACE2) genes has been observed in thyroid tissues, rendering the thyroid more susceptible to the virus [12]. Furthermore, a recent study conducted autopsies on 44 COVID-19 patients, revealing the discovery of the virus within the follicular cells of the thyroid gland, as detected using RNA in situ (RNAscope) detection [11]. Nevertheless, current reviews have summarized existing observational research and suggested that there is still insufficient evidence to establish that thyroid dysfunction directly precipitates viral pneumonia, especially increasing susceptibility to and severity of COVID-19 [12,21,22,23,24,25,26,27]. The majority of extant epidemiological studies are observational, which impose certain limitations [12,21,22,23,24,25,26,27]. Firstly, they struggle to ascertain the chronological sequence of cause and effect [23]. Secondly, the use of thyroid-related medications, anti-COVID-19 drugs, and COVID-19 vaccines can confound the association [21]. However, Mendelian randomization (MR) studies, which analyze phenotypic genetic variations as instrumental variables (IVs), provide a direct causal relationship between exposure and outcomes, all the while being cost-effective [28]. Presently, MR studies have not explored the connection between thyroid function and viral pneumonia.

Hence, to determine whether alterations in thyroid hormone levels, thyroid diseases, and other thyroid dysfunctions can trigger viral pneumonia as well as increase susceptibility to and severity of COVID-19, this study utilizes the MR design to investigate the causal relationship between thyroid function and the risk of viral pneumonia, along with COVID-19.

## 2. Material and Methods

### 2.1. Data Sources

**Figure 1:**
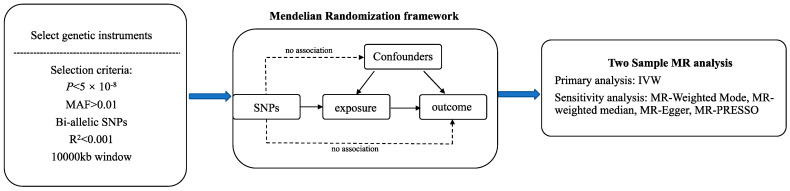
Study Design of the Two-Sample MR method.

In this study, we implemented a two-sample MR model to explore the causal relationships between thyroid function, viral pneumonia, and COVID-19. A comprehensive depiction of the study design can be found in Figure 1. The MR study comprised three primary components: the acquisition of IVs for exposures, the establishment of the MR model, and the utilization of MR methods along with sensitivity analyses [29,30]. In this study, exposures included three thyroid function indicators (thyroid-stimulating hormone (TSH) within reference range, free thyroxine within reference range, TSH within full range) and three thyroid diseases (autoimmune thyroid disease (AITD), subclinical hypothyroidism, subclinical hyperthyroidism). Genetic variants associated with thyroid function were utilized as IVs for the exposure, while summary statistics for viral pneumonia, COVID-19 susceptibility, and COVID-19 severity were considered as outcome data. COVID-19 severity phenotypes encompassed hospitalization due to COVID-19 and critically ill COVID-19 patients [31]. The summary-level data for both exposure and outcome were derived from the largest available Genome-Wide Association Studies (GWASs) [32,33,34,35,36]. To minimize variations related to ancestry, this MR study exclusively focused on individuals of European descent. To assess thyroid function in terms of exposure data, summary statistics were collected from various sources. The ThyroidOmics Consortium provided summary statistics for TSH levels within the reference range, as well as FT4 levels within the reference range, subclinical hypothyroidism, and subclinical hyperthyroidism. Additionally, meta-analyses of existing GWASs were used to extract data on the full range of TSH levels and AITD. Finngen and COVID-19 HGI provided summary-level data for viral pneumonia and COVID-19 as outcome measures. A comprehensive breakdown of the data used in this MR model is presented in Table 1. Ethical approvals and consent information for both the exposure and outcome summary statistics were collected from the original publications.

### 2.2. Instrumental Variable Selection

The process of identifying IVs strictly adhered to the principles of MR design, involving the following criteria:

1. Strong Link to Exposure: Selected IVs were required to exhibit a robust and significant association with the exposure of interest at a genome-wide significance level (*p* < 5 × 10^−8^).
2. Independence from Confounders: IVs needed to be uncorrelated with potential confounding factors to ensure unbiased estimation of causal effects.
3. Exclusive Influence on Outcome via Exposure: IVs should have a sole influence on the outcome through the exposure, ensuring a direct causal relationship [28].

To enhance the accuracy of our MR analysis, we employed linkage disequilibrium pruning to eliminate variants that were highly correlated (R^2^ < 0.001) within a 10 Mb window) with the presumed genetic instruments. We also excluded SNPs with a minor allele frequency <0.01, as they may elevate the risk of type I error and skew the results towards the null. Building upon the findings from Teumer et al.’s GWAS on thyroid function, we identified 61, 31, 8, and 8 independent Single Nucleotide Polymorphisms (SNP) associated with normal-range TSH and FT4 levels, subclinical hypothyroidism, and subclinical hyperthyroidism, respectively [35]. A more recent GWAS meta-analysis unveiled 99 independent top variants across 74 loci, explaining 13.3% of the variance in full-range TSH levels [36]. In the case of AITD, Saevarsdottir et al. reported 99 genome-wide significant associations at 93 independent loci [32]. Nonetheless, several IVs were excluded: (1) TSH within reference range: SNP rs200574439, falling under the category of Insertion and Deletion (InDel), was excluded in this study. The variants Rs8176645 (mapped to ABO) and rs1157994 (mapped to BCAS3) were excluded due to their pleiotropic effects and lack of specificity to thyroid tissue [37]. Additionally, rs12390237 was omitted because it mapped to the X-chromosome, creating disparities as women possess two X-chromosomes, while men have only one X-chromosome [37]; (2) Subclinical Hyperthyroidism: The SNP rs925488 mapped to FOXE1 was discarded because its effect allele was associated with both hypo- and hyperthyroidism simultaneously [37]. Furthermore, several SNPs significantly linked (*p* < 5 × 10^−8^) to potential confounders of the association between thyroid function and viral pneumonia, COVID-19 susceptibility, COVID-19 hospitalization, and COVID-19 critical illness, such as body mass index, smoking, drinking, physical activity, education level, and other respiratory diseases. In order to prevent any potential influence from horizontal pleiotropy, we performed a search using PhenoScanner V2 and excluded these specific SNPs from our analysis [38].

The use of strong instruments is key to improving the accuracy and efficiency of estimating causal effects in the MR model. However, if the genetic variation is only weakly linked to the exposure variable, it may introduce bias in the estimates of the MR model, which is commonly referred to as weak instrument bias [39] We further assessed the strength of genetic variants by computing the F-statistic (F = β^2^/SE^2^) for each SNP, ensuring that the F-statistic exceeded 10 [39,40]. In this study, the minimum F-statistic observed was 29.75, suggesting strong instruments and consequently a low likelihood of bias from weak instruments (Supplementary Table S1).

The extracted summary statistics for the association between thyroid function SNPs and the four outcomes are presented in Supplementary Table S1.

### 2.3. Statistical Analysis

We conducted a comprehensive array of two-sample MR analyses and implemented multiple sensitivity tests to elucidate the causal relationships between thyroid function, viral pneumonia, and COVID-19.

Our primary MR approach was the inverse-variance weighted (IVW) method, which estimated the causal effect of a one-standard deviation increase in genetically predicted exposure on the outcome [41]. In the interest of robustness, we employed several sensitivity analyses, including weighted mode, weighted median, and MR-Egger analyses. Weighted mode regression accommodates situations where over 50% of genetic variants may be invalid [42], while weighted median offers reliable causal estimation when at least 50% of the analysis weight is derived from valid IVs [43]. The MR-Egger approach was applied to compute causal effects in the presence of directional pleiotropy [44]. We conducted assessments for heterogeneity and directional pleiotropy using Cochran’s Q statistics and the MR-Egger intercept test, respectively. Additionally, we employed the Global test and Outlier test in MR-PRESSO analysis to identify horizontal pleiotropy and potential outliers, respectively [45]. If no heterogeneity was detected, we based our main results on a multiplicative random-effects model using IVW; otherwise, a fixed-effects model was employed [40]. In order to strengthen the reliability of the results, this study assessed the statistical power of significant associations. This calculation determined the probability of detecting a true effect in this MR study, taking into account the specified sample size and effect size [46,47]. When two datasets from a prominent global genetics consortium have overlapping studies and participants, it is important to identify the extent of the overlap and evaluate any bias that may arise from it [48]. 

Acknowledging the potential influence of outliers, we performed a secondary analysis in which IVW estimates were recalculated after removing influential SNPs identified through MR-PRESSO analysis.

Our threshold for statistical significance was set at *p* < 0.05. All analyses were carried out using R statistical software (version 4.1.2; R Foundation for Statistical Computing, Vienna, Austria). We leveraged TwoSampleMR (version 0.5.6) and MRPRESSO (version 1.0) for these analyses.

## 3. Results

**Figure 2:**
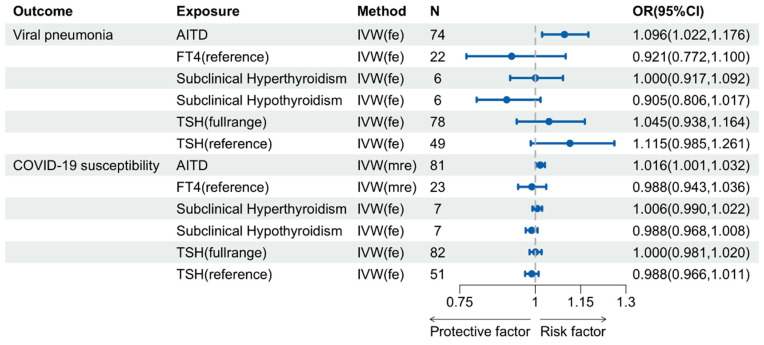
Causal Effects of Thyroid Function on the Incidence Risk of Viral Pneumonia and COVID-19 Susceptibility.

**Figure 3:**
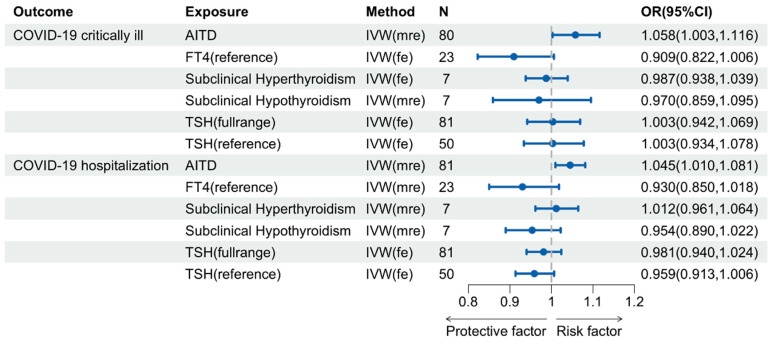
Causal Effects of Thyroid Function on COVID-19 Severity.

A genetic predisposition to AITD displayed significant associations with viral pneumonia (*p* = 0.010) as well as COVID-19 susceptibility (*p* = 0.037), hospitalization (*p* = 0.012), and critical illness (*p* = 0.040) (see Figure 2 and Figure 3). The other three MR methods, including weighted median, weighted mode, and the MR-Egger method, consistently confirmed the direction of the primary method (Supplementary Table S2). Sensitivity analyses unveiled the absence of directional pleiotropy. The Cochran’s Q test and Global test revealed no evidence of heterogeneity and horizontal pleiotropy in the relationship between AITD and viral pneumonia (*P*_Cochran’s Q_ = 0.150; *P*_Global test_ = 0.130). Therefore, this study utilized the fixed-effects IVW model as the primary method to evaluate the causal association between AITD and viral pneumonia. However, both Cochran’s Q test and the Global test identified horizontal, yet balanced pleiotropy in the connection between AITD and the three COVID-19 phenotypes (Supplementary Table S3). Importantly, under the assumption of Instrument Strength Independent of Direct Effect, heterogeneity due to horizontal pleiotropy did not invalidate the IVW estimates automatically [49]. Consequently, transitioning to random-effects IVW models produced estimates equivalent to those in fixed-effects models. The MR analysis in this study utilized the random-effects IVW model to determine the relationship between AITD and susceptibility to, hospitalization due to, and critically ill cases of COVID-19. The results demonstrated a significant association between these factors. The MR PRESSO approach also corroborated the findings of the IVW method and did not detect any outliers (Supplementary Table S3). Despite minor bias due to sample overlap between AITD and COVID-19 hospitalization, our results remained robust, maintaining a type I error rate of 0.05 and a statistical power exceeding 80% (Supplementary Tables S4 and S5). This provides strong evidence for the positive associations.

In contrast, there was insufficient evidence to support a causal impact of TSH (reference/full range), FT4, subclinical hypo- or hyperthyroidism on viral pneumonia and COVID-19 (see Figure 2 and Figure 3).

The sensitivity analysis did not find any evidence of directional pleiotropy, but it did identify heterogeneity in six different models. These models evaluated the relationships between COVID-19 susceptibility and FT4 levels, COVID-19 susceptibility and TSH levels within the reference range, COVID-19 hospitalization and subclinical hyperthyroidism, COVID-19 hospitalization and FT4 levels, COVID-19 hospitalization and subclinical hypothyroidism, and COVID-19 critically ill patients and subclinical hypothyroidism. Within these six models, four of them (COVID-19 susceptibility and FT4 levels, COVID-19 hospitalization and FT4 levels, COVID-19 hospitalization and subclinical hypothyroidism, COVID-19 critically ill patients and subclinical hypothyroidism) displayed horizontal pleiotropy, as determined by the Global test (Supplementary Table S3). In the subsequent three models (COVID-19 hospitalization and FT4, COVID-19 hospitalization and subclinical hypothyroidism, COVID-19 critically ill patients and subclinical hypothyroidism), outliers were detected, and MR analysis was rerun (Supplementary Table S3). As expected, the results after removing the outliers remained consistent with the null hypothesis of the primary findings (Supplementary Table S6). Importantly, it was observed that the sample overlap rates between TSH (reference/full range), FT4, subclinical hypo- or hyperthyroidism, and the four outcomes were all significantly less than 10%, thereby minimizing the impact of bias on the results (Supplementary Table S4).

## 4. Discussion

The findings of this study, utilizing MR analysis, indicate a cause-and-effect relationship between AITD and a heightened risk of both viral pneumonia development and susceptibility to COVID-19. Furthermore, upon closer examination of AITD’s impact on the severity of COVID-19, it was revealed that AITD also elevates the risk of hospitalization and critical illness resulting from COVID-19. However, no significant causal relationship was established between individual thyroid hormone markers, subclinical hypothyroidism, hyperthyroidism, and the risk of viral pneumonia or COVID-19.

This MR study is pioneering in its exploration of the influence of thyroid function on viral pneumonia. Among the diverse thyroid function statuses and their associations with the incidence risk of viral pneumonia, we have discerned a causal link between AITD and viral pneumonia. The summary statistics related to viral pneumonia in this MR analysis were sourced from the most recent release of FinnGen results, ensuring a lack of sample overlap between exposure and outcome [33]. During the IV selection process, we meticulously excluded SNPs significantly associated with viral pneumonia and evaluated the F-statistics of the SNPs to ensure the exclusion of weak IVs. Furthermore, we utilized the Phenoscanner website to detect and remove any genetic variants that may be associated with other factors that could potentially interfere with or distort the true causal relationships [38]. In our primary analysis, we applied the fixed-effect IVW method to establish the association between AITD and viral pneumonia. The IVW method offers the highest statistical power when the validity requirements of the MR key assumptions are met. In subsequent sensitivity analyses, no heterogeneity, directional pleiotropy, horizontal pleiotropy, or outliers were detected. Moreover, the statistical power exceeded 80%, reinforcing the significant association between AITD and viral pneumonia [49]. Combining the primary and sensitivity analyses, the results from the IVW method demonstrate that AITD causally impacts viral pneumonia, without any signs of heterogeneity or pleiotropy. Turning to the outcomes related to COVID-19, this MR study explored both susceptibility and severity resulting from AITD. Similarly, we observed that AITD causally increases susceptibility and severity in the context of COVID-19. For this investigation, we utilized the seventh round of the COVID-19 GWAS released by the Human Genetics of Infectious Diseases (HGI), which currently stands as the largest available dataset. Acknowledging the existence of heterogeneity, we employed the random-effects IVW method to establish the connection between AITD and the susceptibility and severity of COVID-19. In subsequent sensitivity analyses, the global test of MR PRESSO indicated the presence of horizontal pleiotropy, but no outliers were detected. Despite the overlap between AITD and COVID-19, the bias introduced through sample overlap did not alter the association between AITD and COVID-19 susceptibility and severity. The MR analyses assessing the impact of AITD on COVID-19 benefited from substantial statistical power, with the power exceeding 80%.

While the exact mechanism through which AITD impacts the risk of viral pneumonia and COVID-19 remains not fully elucidated, it is well established that immunocompromised individuals are at higher risk for developing severe infections during respiratory viral illnesses [50,51]. Fung et al. summarized that immunocompromised patients have a higher risk of contracting severe COVID-19 and experiencing increased mortality [52]. In AITD, the increase in Th22 cells, which play a role in the development of various autoimmune diseases, and the resultant imbalance of the immune system can compromise individual immune function [17,53,54]. This may also be a contributing factor to the increased vulnerability of AITD patients to viral pneumonia. Furthermore, in the presence of AITD, the expression of S1PR1 is elevated in T CD4+ cells. This, in turn, leads to the activation of STAT3 signaling through two cascades: S1PR1/mTOR/PSer^727^STAT3 and S1PR1/JAK2/PTyr^705^STAT3 cascades [55]. In turn, STAT3 recurrently activates the expression of S1PR1, creating a positive feedback regulatory mechanism. This results in an increased production of interleukin-6 (IL-6) [56]. In addition, IL-6 also triggers the increased expression of cathepsin L and ACE2 receptors, leading to a higher viral load and an increased risk of lung injury [57]. It is worth mentioning that the elevated levels of cytokines associated with AITD may have a significant impact on the levels of molecules that mediate SARS-CoV-2 infection. This could potentially increase the susceptibility to COVID-19. Zhao et al. made a finding that the introduction of different levels of AITD-related cytokines leads to increased levels of interferon-γ (IFN-γ) and tumor necrosis factor-α (TNF-α), which in turn enhance the expression of ACE2 and NRP1 [58]. ACE2 and NRP1 serve as established receptors that facilitate the entry and infection of SARS-CoV-2 [59,60,61]. Therefore, higher levels of IFN-γ and TNF-α observed in patients with AITD might contribute to a greater vulnerability to SARS-CoV-2 infection in the thyroid. This is achieved by increasing the levels of ACE2 and NRP1. 

In contrast, the results of this MR study did not provide significant evidence of a causal relationship between thyroid hormone levels, subclinical hypo- or hyperthyroidism, and the development of viral pneumonia or the incidence and severity of COVID-19. These findings are in alignment with previous cross-sectional studies [12,62,63,64], indicating that alterations in isolated thyroid function indicators alone may not be sufficient to reliably predict the development of viral pneumonia or COVID-19. Prior to this study, four MR investigations examined the relationship between thyroid hormones and COVID-19, utilizing data from the fifth round of the Human Genetics of Infectious Diseases (HGI) releases [31,65,66,67]. In 2023, Sun et al. employed an MR model to assess the causal link between TSH levels and three COVID-19 phenotypes and concluded that there was no association between TSH and COVID-19 [65], a conclusion consistent with our research. Compared to previous studies, our analysis utilized data from the seventh round, which had a larger sample size than the fifth round. However, this round exhibited sample overlap between exposures and outcome data. To assess the impact of sample overlap, this study calculated the bias and Type I error using an online tool designed by Burgess et al. [48]. The results indicated that the bias stemming from sample overlap between COVID-19 susceptibility and critical illness and thyroid phenotypes was negligible (0), with a Type I error rate of 0.05, suggesting that sample overlap did not substantially affect the causal results [48,68]. Regarding AITD-COVID hospitalization, our study identified a minor bias of 0.012 caused by sample overlap. However, it did not lead to false-positive findings (Type I error rate = 0.05). Given the use of robust IVs in the MR analysis of AITD-COVID hospitalization, we maintain confidence in the positive association between AITD and COVID hospitalization. Although using the fifth round of data with non-overlapping samples can eliminate sample overlap, a drawback is the potential loss of efficiency if the estimation of genetic associations is less accurate [48,69].

This study presents several strengths. Firstly, it stands as the pioneering MR investigation to establish a causal link between thyroid function and the development of viral pneumonia. MR study sheds light on possible cause-and-effect relationships between exposure and disease outcomes, while also reducing the time and effort required for long-term follow-up [70]. By utilizing available extensive GWAS data, MR studies can be conducted in a more efficient and affordable manner. Secondly, the genetic instruments selected to assess thyroid function in this research demonstrate robustness. The MR method serves as a statistical model of genetic variation, acting as an IV. The generation of genetic variation is not influenced by factors such as social environment, lifestyle habits, or other traits [28]. Moreover, genetic variation exists before the occurrence of environmental exposures, confounders, and diseases. By using genetic variation as an IV for exposure, it ensures that the variation in exposure is explained by genetic factors which come before the outcomes. This effectively eliminates the problem of reverse causality [71]. In this study, we strictly adhered to the three principles of MR when selecting IVs and computing their F-statistics. Thirdly, a rigorous screening process was applied to eliminate potential confounding factors, enhancing the reliability of our MR results. Sensitivity analysis not only encompassed additional MR methods beyond the primary analysis but also meticulously scrutinized and quantified levels of heterogeneity and pleiotropy. Additionally, in order to detect and account for the influence of outliers on the primary results, MR PRESSO was deployed to identify such outliers, and a secondary analysis was conducted accordingly. Moreover, this study calculated statistical power and assessed biases originating from sample overlap, thus reinforcing the comprehensiveness and rigor of our MR investigation. The GWAS data used for both exposure and outcome were sourced from the most extensive available datasets, further bolstering the reliability of the study’s findings.

Nonetheless, this study is not without limitations. Firstly, while this study revealed a causal relationship between AITD and the development of viral pneumonia and COVID-19, it is crucial to acknowledge that the absence of GWAS data for other types of thyroid diseases, such as acute/subacute thyroiditis or drug-induced thyroiditis, prevents us from ascertaining whether these other forms of thyroid diseases have similar impacts on viral pneumonia and COVID-19. Furthermore, given that two-sample MR studies necessitate exposure and outcome data from the same ancestral background, this study exclusively focused on the European population. Therefore, further research is imperative before extending the results to other population groups. This study encountered challenges in constructing a robust multivariable MR model due to the inadequate strength of the IVs. The model was significantly influenced by the bias introduced by the weak IVs [72]. Considering the complex regulatory connections among diabetes, hypertension, obesity, and thyroid function, it is advisable for future research to explore alternative models. In order to gain a better understanding of this intricate interaction, it is crucial to conduct a thorough study using specific research methodologies. These new studies will facilitate an in-depth exploration of the regulatory effects on thyroid function indicators, viral pneumonia, and COVID-19. It will also help uncover the complex cause-and-effect relationships among these factors. While this study supports the notion that AITD contributes to the development of viral pneumonia and exacerbates the susceptibility and severity of COVID-19, it is essential to recognize that the prevention and treatment of COVID-19 may require a deeper understanding of the specific pathways associated with its development. Additional research and validation are still required to elucidate these mechanisms.

## 5. Conclusions

This two-sample MR analysis revealed a significant positive association between AITD and the risk of viral pneumonia, as well as the susceptibility and severity of COVID-19. As a result, it is imperative to intensify health education for AITD patients and bolster health monitoring for individuals who have contracted viral pneumonia or COVID-19, especially those with a predisposition to AITD. Future research should delve deeper into the potential specific pathway mechanisms that underlie how AITD elevates the risk of viral pneumonia and COVID-19. This pursuit of knowledge will be instrumental in informing more effective strategies for the prevention and management of these conditions.

## Figures and Tables

**Table 1:** Data Sources for MR Analysis.

Phenotype	Consortium	Definition	Ample Size
AITD [32]	Iceland, UK Biobank	Individuals who had received a diagnosis of Graves’ disease or Hashimoto’s thyroiditis were considered cases of AITD as well as those who had been diagnosed with other hypothyroidism and/or had received thyroxin treatment, excluding known non-autoimmune causes of hypothyroidism (thyroid cancer, drug-induced hypothyroidism, amiodarone and interferon treatments).	Cases = 30,234; Controls = 725,172
Viral pneumonia [33]	Finngen (Release 9)	Inflammation of the lung parenchyma that is caused by a viral infection.	Cases = 3394; Controls = 314,673
COVID-19 critically ill [34]	COVID-19 HGI (Round 7)	Very severe patients, defined as patients who died or required respiratory support (including continuous positive airway pressure, bilevel positive airway pressure, intubation, or high- flow nasal cannula)	Cases = 13,769; Controls = 1,072,442
COVID-19 hospitalization [34]	COVID-19 HGI (Round 7)	Patients who were hospitalized for COVID-19	Cases = 32,519; Controls = 2,062,805
COVID-19 susceptibility [34]	COVID-19 HGI (Round 7)	Patients with COVID-19 defined as individuals with laboratory confirmation of SARS-CoV-2 infection or electronic health records or self-reported	Cases = 122,616; Controls = 2,475,240
Subclinical hypothyroidism [35]	ThyroidOmics	Cases defined on the basis of TSH level above the reference range, but without overt thyroid disease (thyroid surgery or medication use)	Cases = 3440; Controls = 49,983
Subclinical hyperthyroidism [35]	ThyroidOmics	Cases defined on the basis of TSH level below the reference range, but without overt thyroid disease (thyroid surgery or medication use)	Cases = 1840; Controls = 49,983
TSH(reference) [35]	ThyroidOmics	TSH within cohort-specific reference range without overt thyroid disease (thyroid surgery or medication use)	54,288
TSH(full range) [36]	ThyroidOmics, MGI, HUNT study	ThyroidOmics: individuals without records of thyroid medication or surgery MGI: individuals with any thyroid disorders were excluded based on electronic records HUNT study: individuals with any thyroid disorders were excluded based on self-report, blood tests indicating clearly overt hypothyroidism, and cancer registry data	119,715
FT4(reference) [35]	ThyroidOmics	FT4 within cohort-specific reference range without overt thyroid disease (thyroid surgery or medication use)	49,269

Abbreviation: MGI: Michigan Genomics Initiative; HUNT: Nord-Trøndelag Health Study; HGI: host genetics initiative.

## Data Availability

The data presented in this study are openly available in Table 1.

## Supplementary Materials

The following supporting information can be downloaded at: https://www.mdpi.com/article/10.3390/pathogens13020101/s1, Table S1: Information of Selected Instrument Variables; Table S2: Results of MR Analysis; Table S3: Results of Sensitivity Analysis; Table S4: Sample Overlap Bias; Table S5: Power of MR Analysis; Table S6: Results after Outlier Removal.
